# Bioinformatic analysis of structures and encoding genes of *Escherichia coli* surface polysaccharides sheds light on the heterologous biosynthesis of glycans

**DOI:** 10.1186/s12864-023-09269-6

**Published:** 2023-04-04

**Authors:** Ao Dong, Chengzhi Liu, Xiaoting Hua, Yunsong Yu, Yan Guo, Dongshu Wang, Xiankai Liu, Huan Chen, Hengliang Wang, Li Zhu

**Affiliations:** 1 State Key Laboratory of Pathogen and Biosecurity, Beijing Institute of Biotechnology, No. 20, Dongda Street, Fengtai District, Beijing, 100071 People’s Republic of China; 2 Department of Infectious Diseases, Sir Run Run Shaw Hospital, Zhejiang University School of Medicine, Hangzhou, People’s Republic of China; 3 Hangzhou Digital-Micro Biotech Co., Ltd, Hangzhou, People’s Republic of China; 4 Key Laboratory of Microbial Technology and Bioinformatics of Zhejiang Province, Hangzhou, People’s Republic of China; 5 Regional Medical Center for National Institute of Respiratory Diseases, Sir Run Run Shaw Hospital, Zhejiang University School of Medicine, Hangzhou, People’s Republic of China; 6 Zhejiang Chinese Medical University, Hangzhou, People’s Republic of China

**Keywords:** *Escherichia coli*, Surface polysaccharide, Glycosyltransferase, Serotype, Synthetic glycobiology

## Abstract

**Background:**

Surface polysaccharides (SPs), such as lipopolysaccharide (O antigen) and capsular polysaccharide (K antigen), play a key role in the pathogenicity of *Escherichia coli* (*E. coli*). Gene cluster for polysaccharide antigen biosynthesis encodes various glycosyltransferases (GTs), which drive the process of SP synthesis and determine the serotype.

**Results:**

In this study, a total of 7,741 *E. coli* genomic sequences were chosen for systemic data mining. The monosaccharides in both O and K antigens were dominated by D-hexopyranose, and the SPs in 70–80% of the strains consisted of only the five most common hexoses (or some of them). The linkages between the two monosaccharides were mostly α-1,3 (23.15%) and β-1,3 (20.49%) bonds. Uridine diphosphate activated more than 50% of monosaccharides for glycosyltransferase reactions. These results suggest that the most common pathways could be integrated into chassis cells to promote glycan biosynthesis. We constructed a database (EcoSP, http://ecosp.dmicrobe.cn/) for browse this information, such as monosaccharide synthesis pathways. It can also be used for serotype analysis and GT annotation of known or novel *E. coli* sequences, thus facilitating the diagnosis and typing.

**Conclusions:**

Summarizing and analyzing the properties of these polysaccharide antigens and GTs are of great significance for designing glycan-based vaccines and the synthetic glycobiology.

**Supplementary Information:**

The online version contains supplementary material available at 10.1186/s12864-023-09269-6.

## Introduction

*Escherichia coli* (*E. coli*) consists of commensal and pathogenic strains, some of which can cause serious infectious diseases. Polysaccharides are important extracellular polymeric substances of *E. coli*. They usually maintain their physical and chemical properties and the biological functions of cells, thus playing a protective role in bacteria. Among them, lipopolysaccharide (LPS) and capsular polysaccharide (CPS) have specific immunogenicity and are strongly associated with the pathogenicity of *E. coli* [1–3].

Compared with Gram-positive bacteria, Gram-negative bacteria have a thinner peptidoglycans layer but can generate an additional outer membrane with LPS as the main component [4, 5]. LPS is a large glycolipid consisting of three domains, including lipid A, core oligosaccharide, and O antigen, and the difference in the O antigen is the main reason for LPS diversity [6, 7]. O antigen exists on the surface of bacteria, has high immunogenicity, and is an important virulence factor involved in the immune escape process of bacteria [2, 8, 9]. The outer side of *E. coli* is usually surrounded by a large amount of CPS, the structural basis of its biological adhesion. It is generally believed that CPS attenuates the complement-mediated immune process and leads to immune escape [10–12]. Therefore, LPS and CPS are the main immune targets of *E. coli*. Many vaccines based on these surface polysaccharides (SPs) have been developed [13–15]. Several studies have further shown that CPS and LPS can be used as combined targets to obtain more effective vaccines [16, 17].

The structure of O antigens is diverse, with variations in chain length, the sugar residue, or glycosidic bond among different serotypes [7, 18, 19]. However, from the perspective of the general synthesis process, there are still high similarities between different strains. The O antigen is synthesized progressively on a lipid carrier molecule, undecaprenyl phosphate, in the cytoplasm of the bacterial cell, and it is then transferred to the periplasm and linked with the core oligosaccharide to constitute nascent LPS and release the lipid carrier [20, 21]. The O antigen sugar chain synthesis processes are significantly different, usually through the Wzy-dependent pathway, ABC-dependent pathway, or synthase-dependent pathway [6, 19]. The synthesis process of CPS is still not fully defined, but previous studies have shown that it is also involved in the above three pathways [10, 22].

Enzymes encoded by antigen-synthesis genes are core drivers in the synthesis and assembly of SPs. These genes are mainly classified into three categories [7, 10, 11, 23]: nucleotide-sugar precursor synthesis, glycosyltransferase (GT), and antigen unit processing genes. The enzymes encoded by these genes catalyze the synthesis pathway from monosaccharides to complete antigens; so, combining various genes is essential to study serotypes. By summarizing and analyzing the corresponding relationship between each serotype and polysaccharide antigen structure, synthetic gene cluster, polysaccharide synthesis information, monosaccharide synthesis pathway, strain genome, and strain name, we constructed a database of polysaccharide antigen structure of *E. coli*. Based on the database, an online platform (*Escherichia coli* Surface Polysaccharide, EcoSP) was developed for information retrieval and typing analysis of *E. coli* polysaccharide antigens. It is significant for clinical diagnosis, microbial resource development, vaccine development, and epidemiological investigation.

## Materials and methods

### Information collection of strains and genomes

The genome assemblies of all *E. coli* strains deposited in the NCBI database were downloaded (June 5, 2021). Then, we evaluated the quality of those assemblies using CheckM [24] and FastANI [25]. Subsequently, the data were strictly screened to eliminate the low-quality strain according to the following filter criteria: numbers of genomic fragments < 500, genomic integrity > 85%, gene contamination < 5%, and harboring the sugar antigen gene cluster (the identity and coverage with reference sequences of O or K-antigen clusters [7, 26] are greater than 90% and 60% respectively). In addition, the related information of these qualified strains was also retrieved (such as strain ID, submission date, and geographical origin) or analyzed (such as annotations for polysaccharide synthesis genes and predicted serotypes).

### Information collection of antigens

Antigen information was mainly collected from published literature and combined with NCBI, MetaCyc [27] and GTDB [28], including the antigen structures, antigen-synthesis gene clusters, and gene sequences. The CSDB/SNFG structure editor [29] (http://csdb.glycoscience.ru/snfgedit/snfgedit.html), which can visualize the oligosaccharide chain by using Symbol Nomenclature for Glycans (SNFG), was used to draw the structure of each antigen based on the relevant information. The enzymes encoded by the antigen-synthesis genes were also recorded with their relevant information. The information of donors, acceptors, and glycosidic bonds was linked to GTs, while the information of precursors, products, and synthetic pathways was linked to nucleotide-sugar precursor synthase. All strains were linked to the antigen information and each specific serotype derived from the process described above; so, the contained information became a knowledge graph. Each screened information was manually checked. The validated *E. coli* O antigen gene cluster sequences were used as the reference sequences for identifying the serotype of the uploaded sequence.

### Data management and utilization

For easy storage and access, these data were organized into a database. Additionally, effective analysis tools were also developed. Several website modules were constructed to explore the database, including antigenic type browsing, antigenic type retrieval, polysaccharide synthesis retrieval, monosaccharide synthesis retrieval, and sequence upload and analysis modules. Gene prediction of the user-uploading strain genome was performed by Prodigal [30]. Basic Local Alignment Search Tool (BLAST) was applied to compare the input sequence with the reference sequence in the database to predict GTs and serotype (blastx method, identity > 80% & e value < 10^–5^).

## Results

### Strain characteristics

A total of 7,741 *E. coli* genome assemblies were collected in this study. These assemblies date back to 2004, with a significant increase in number since 2014, which may be strongly related to the continuous development of sequencing techniques (Figure S1). The epidemiological study of *E. coli* is becoming mature, and the relevant information for each strain has substantially improved.

**Fig. 1:**
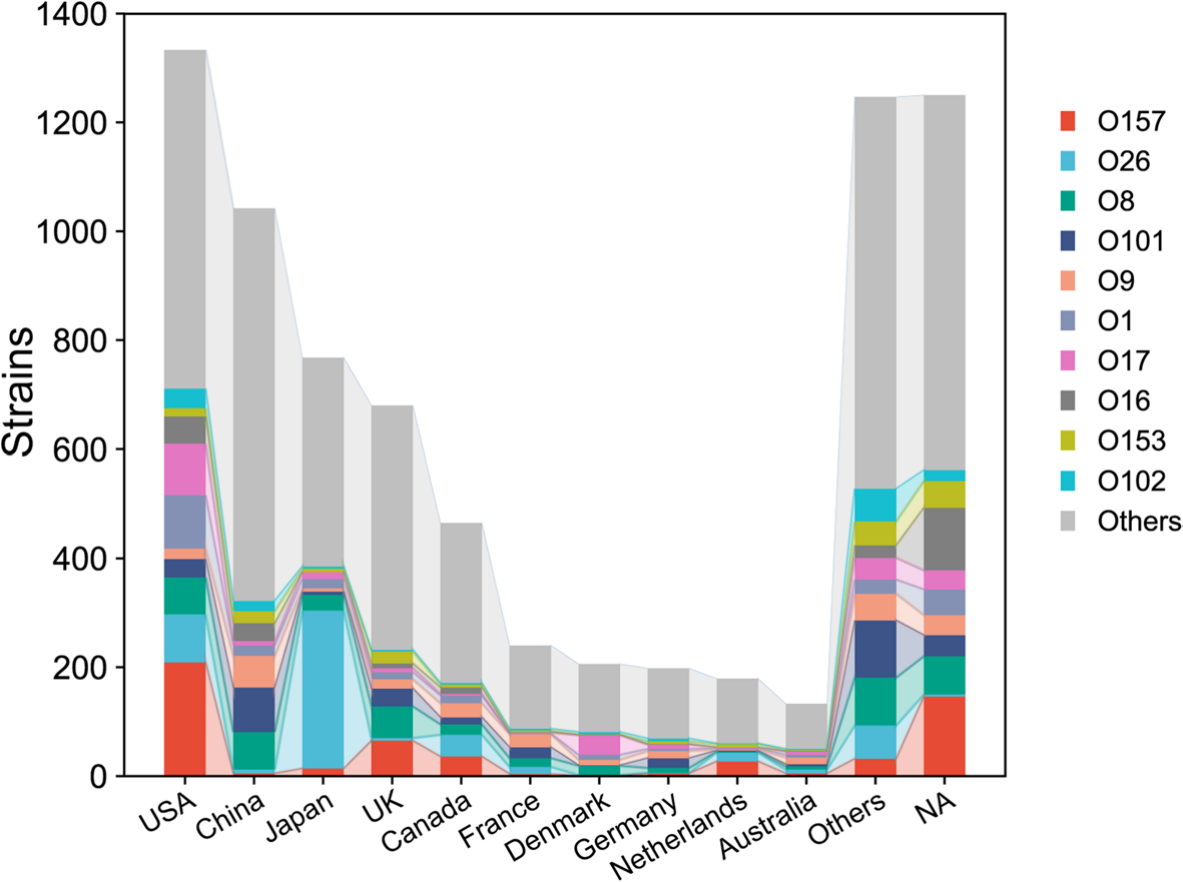
Serotype composition in different countries. Composition of the 10 most common O antigen serotypes in the countries with the most strains

Currently, strains with available genome sequences were mainly isolated from East Asia, North America, or Europe (Figure S2). These sources of information are biased, as the quality of the scientific research will significantly affect their ability to obtain strain information. Despite this inevitable drawback, the information is an important indicator for epidemiological study. We classified the strains according to their serotypes and found that the prevailing serotype significantly differed among countries. The number of the top 10 serotypes included is relatively balanced in the United States, while O26 serotype strains account for more than one-third of the total strains in Japan (Fig. 1). The antigen of the O26 serotype, composed of only glucose (Glc), fucose (Fuc), and rhamnose (Rha), may confer a significant survival advantage on the corresponding strain, which contributed to increasing outbreaks in Japan [31]. Due to the diversity in prevailing serotypes between different regions, region-specific studies are necessary for vaccine development.

### Sugar chain characteristics of antigens and corresponding glycosyltransferase

Data on the structure of 186 O antigens and 68 K antigens were collected from the published literature. In addition, the information on the antigen-synthesis gene cluster and corresponding gene sequence deposited in the NCBI database was available for the O antigen. However, for the K antigen, this information was mostly unavailable.

Most sugar chain structures are formed by different combinations of limited types of sugars, which GTs mainly determine. Because of their diversity and important role in the biosynthesis pathway of *E. coli* SPs, we have gathered information on the GTs, including their donors, acceptors, and glucosidic bonds. This information contained 491 enzymatic reactions. Among them, the enzymes of 79 reactions are not clear, and the other 412 reactions involve 265 GTs or polymerases.

**Fig. 2:**
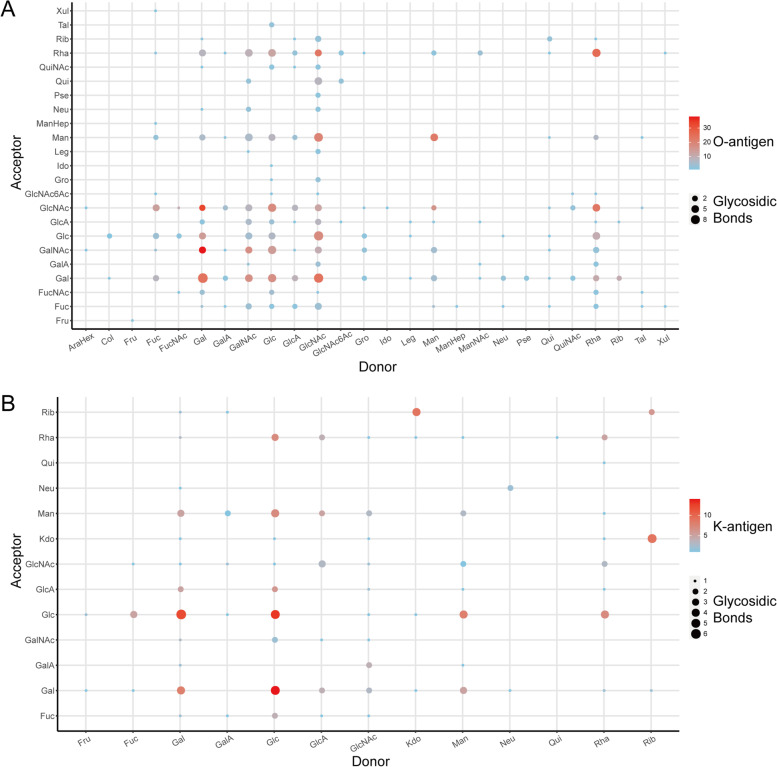
Frequency of donor and acceptor among different antigens. The location of each dot indicates the donor and acceptor of a linkage, its size indicates the number of link types, and the color indicates its frequency of occurrence in O antigens (**A**) or K antigens (**B**). Abbreviations: *AraHex* Arabino-hexose, *Col* Colitose, *Fru* fructose, *Fuc* Fucose, *FucNAc* N-acetylfucosamine, *Gal* Galactose, *GalA* Galacturonic acid, *GalNAc* N-acetylgalactosamine, *Glc* Glucose, *GlcA* Glucuronic acid, *GlcNAc* N-acetyl glucosamine, *GlcNAc6Ac* 6-acetyl N-acetyl glucosamine, *Gro *Glycerol, *Kdo* 3-deoxy-d-manno-oct-2-ulosonic acid, *Ido* Iduronic acid, *Leg* Legionaminic acid, *Man* Mannose, *ManHep* Manno-heptose, *ManNAc* N-acetyl mannosamine, *Neu* N-acetylneuraminic acid, *Pse* Pseudaminic acid, *Qui* Quinovose, *QuiNAc* N-acetyl quinovosamine, *Rha* Rhamnose, *Rib* Ribitol, *Tal* Talose, *Xul* Xylulose

The collected sugar chain structures of these serotypes were compared to study the similarities and differences between each other. As for the O antigen, the glycosidic bonds involving galactose (Gal), GalNAc, Glc or GlcNAc are particularly common, and notably, they occur frequently in various antigens or strains with many different types of linkages (Fig. 2A). Connections with mannose (Man) or Rha are common too, while connections with other sugars are less common, occurring mostly in branched chains or certain serotypes. The types of sugars involved in the K antigen carbohydrate chain are slightly fewer than that in the O antigen (Fig. 2B). The links involved in the aforementioned four sugars are also important in K antigen, but unlike O antigen, ribitol (Rib) or 3-deoxy-d-manno-oct-2-ulosonic acid (Kdo) links also contribute to many K antigen structures.

**Fig. 3:**
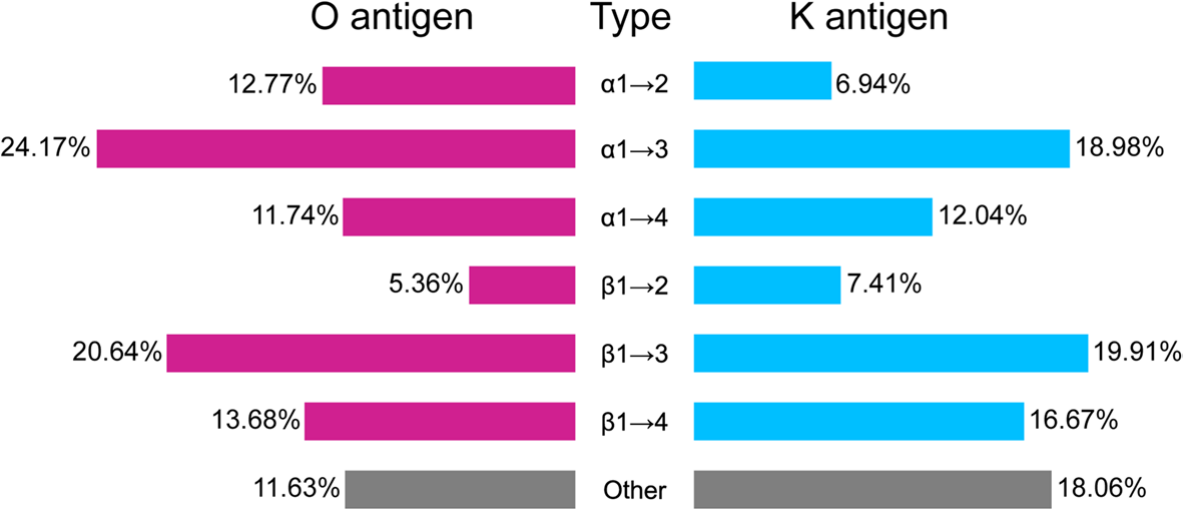
Related carbon site of donor or acceptor in the glycosidic bond. The proportion of each related carbon site in all sugar chain structures

**Fig. 4:**
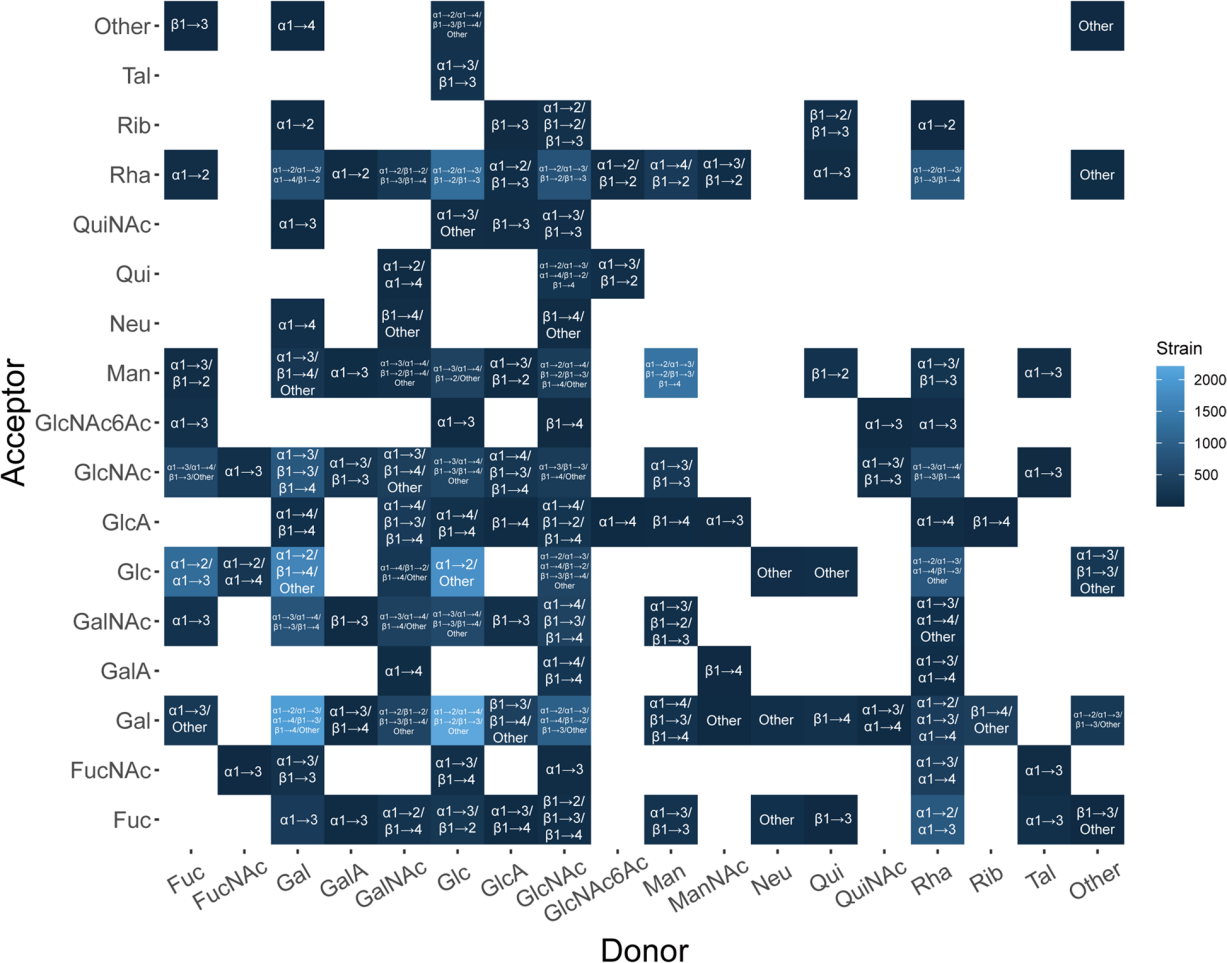
Glycosidic bonds among the O antigen in different strains. The color of each tile indicates the frequency of donor–acceptor connections in different strains. The linkage types have been labeled, including the six common glycosidic bonds and others. Abbreviations: *Fuc* Fucose, *FucNAc* N-acetylfucosamine, *Gal* Galactose, *GalA* Galacturonic acid, *GalNAc* N-acetylgalactosamine, *Glc* Glucose, *GlcA* Glucuronic acid, *GlcNAc* N-acetyl glucosamine, *GlcNAc6Ac* 6-acetyl N-acetyl glucosamine, *Man* Mannose, *ManNAc* N-acetyl mannosamine, *Neu* N-acetylneuraminic acid, *Qui* Quinovose, *QuiNAc* N-acetyl quinovosamine, *Rha* Rhamnose, *Rib* Ribitol, *Tal* Talose

The linkage type of the SP showed some preferences (Fig. 3). In the glycosidic bond of the O antigen, the donor sugar mainly uses its 1-C, both α- and β- anomeric form, while the acceptor sugar is mainly connected to its 2-C, 3-C, or 4-C. Therefore, there are six common glycosidic bonds, α1 → 2, α1 → 3, α1 → 4, β1 → 2, β1 → 3, and β1 → 4, in O antigens. We hypothesized that this might be because C1 is the most activated carbon; so, the 1-C of the donor is more likely to be connected to the receptor. However, the 1-C of the receptor may connect to the previous sugar, which causes the receptor to be connected to other carbon. The overall situation is similar in K antigen, but several donor sugars will be linked by their α2-C site, which is Kdo or N acetylneuraminic acid (Neu). In particular, we refined the glycosidic bond information of O antigen based on strain information. The connections of Gal-Gal, GalNAc-Gal, GalNAc-Man, Glc-Gal, Glc-GalNAc, Glc-GlcNAc, GlcNAc-Gal, GlcNAc-Glc, GlcNAc-Man, GlcNAc-Qui, Man-Man, and Rha-Glc all contain more than 4 common glycosidic bonds (Fig. 4). In addition, the Glc-Gal connection is widely distributed in more than 2000 strains. Some sugars might be connected in a fixed manner. For example, when talose (Tal) is used as a donor, it usually connects to the acceptor by α1 → 3 type.

### Classification of sugars in surface polysaccharides

Monosaccharides are the material basis of antigen synthesis; some have several modifications. The types of monosaccharides differ between O antigen and K antigen.

**Fig. 5:**
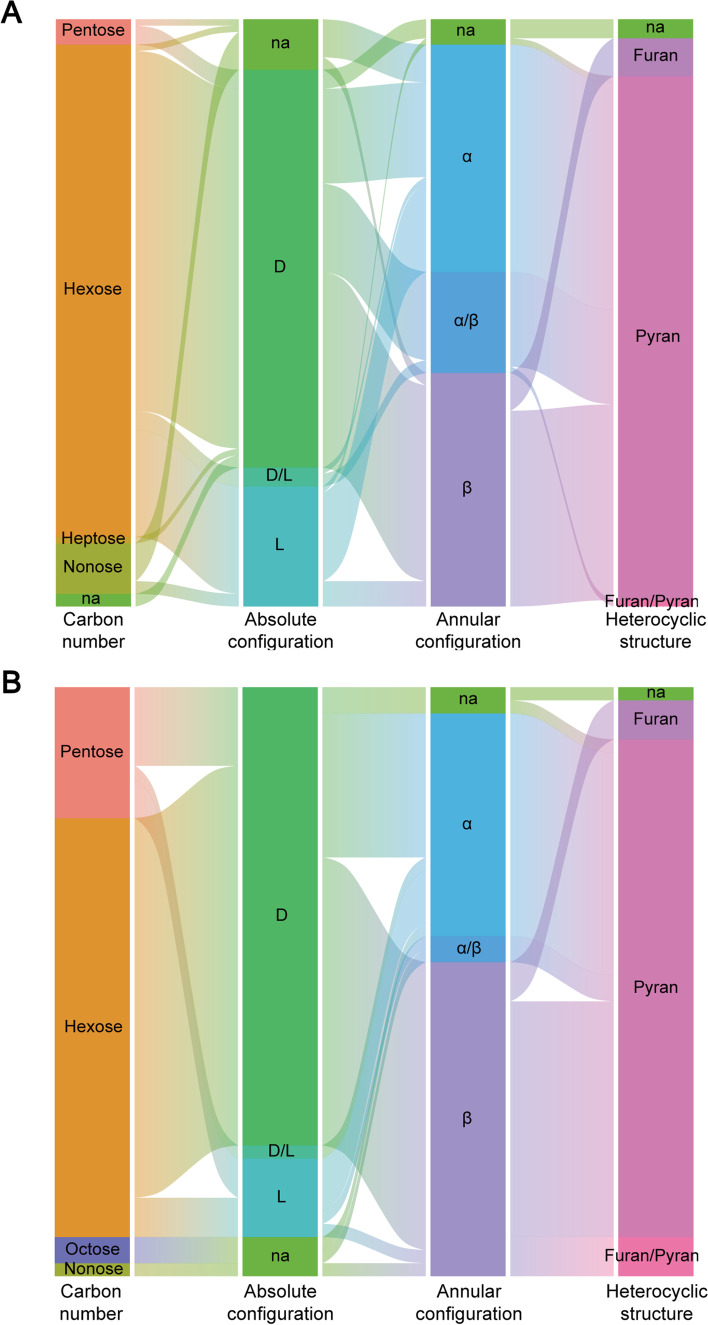
Composition of sugars under different classifications in O antigen and K antigen. (**A**) Composition in O antigen. (**B**) Composition in K antigen. According to the number of carbons, sugars can be named pentose, hexose, heptose, octose, and nonose, among others. Furthermore, a sugar is divided into α/β or D-/L- according to their configuration and pyran or furan according to the heterocyclic structure

O antigen structure is based on combining a total of 113 monosaccharides. In terms of the carbon number, hexoses account for the majority, but there are also a certain number of pentoses, heptoses, and nonoses (Fig. 5A). These monosaccharides are predominantly of the pyranose form, consistent with the fact that the pyranose form is the preferred conformation of natural monosaccharides in an aqueous solution. The total number of saccharides with α- or β- configuration is similar, although different monosaccharides prefer different anomeric forms. For example, α anomer is the predominant form of mannose. Some saccharides possess both α- and β- anomeric forms. There are more D-saccharides than L- saccharides in the antigen, except for a small percentage of saccharides with both D- and L- configurations. This is mainly due to the different abundance in nature, among which most monosaccharides are in the D- configuration, and the L- configuration is converted from D-. There are only 45 monosaccharides found in the K antigen. Among them, hexose is still predominant, pentose is utilized more frequently than in O antigen, and nonose is more infrequent (Fig. 5B). There is no heptose, but there are several octoses. Moreover, absolute configuration, annular configuration, and heterocyclic structure of the K antigen are quite similar to that of the O antigen, except for an increase in the proportion of sugars that have both pyran and furan structures and a decrease in the proportion of sugars that have both α- and β- anomeric forms.

**Fig. 6:**
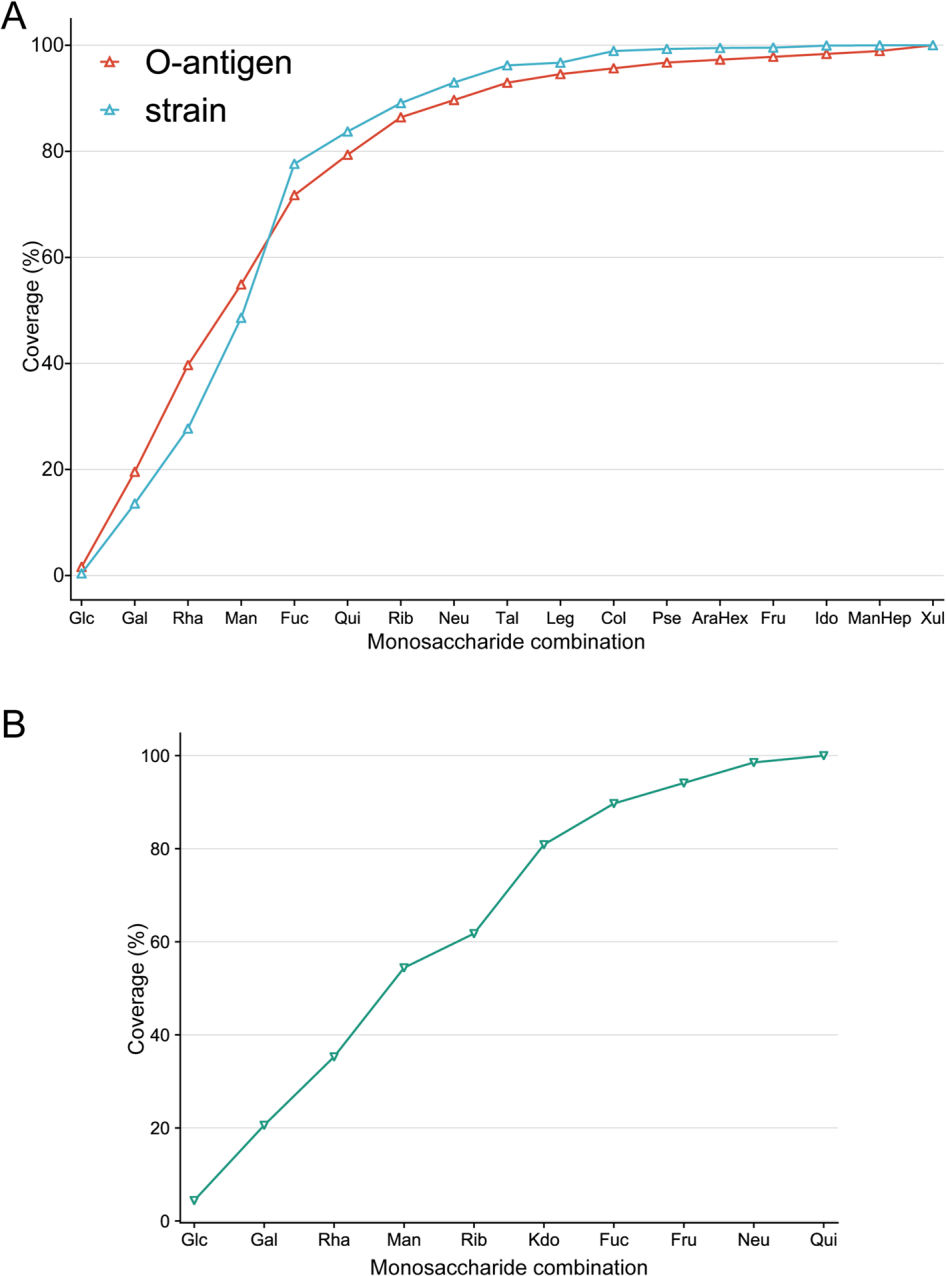
Coverage of monosaccharide combination on sugar chain structure. The abscissa represents the combination of monosaccharides from the far left to that position. (**A**) Ordinate indicates the percentage of O antigens or strains the combination can cover fully. (**B**) Ordinate indicates the percentage of K antigens the combination can fully cover. Abbreviations: *AraHex* Arabino-hexose, *Col* Colitose, *Fru* Fructose, *Fuc* Fucose, *Gal* Galactose, *Glc* Glucose, *Ido* Iduronic acid, *Kdo* 3-deoxy-d-manno-oct-2-ulosonic acid, *Leg* Legionaminic acid, *Man* Mannose, *ManHep* Manno-heptose, *Neu* N-acetylneuraminic acid, *Pse* Pseudaminic acid, *Qui* Quinovose, *Rha* Rhamnose, *Rib* Ribitol, *Tal* Talose, *Xul* Xylulose

Because the biological synthesis of O antigens is well studied, we further explored the sugar composition in O antigens. The occurrence frequency of each monosaccharide was sorted from high to low to discover the least combination of monosaccharides with the highest coverage for strains and antigens (Fig. 6A). We found that the combination of five sugars (Glc-Gal-Rha-Man-Fuc) would cover more than 70% of O antigen or strains. On this basis, adding four sugars (quinovose-Rib-Neu5Ac-Tal) can increase the coverage to nearly 93% and 96% for O antigens and strains, respectively. Other monosaccharides are mainly present in some specific serotypes, which account for 7.07% of all serotypes and 3.79% of strains. Therefore, the synthetic pathways for five activated monosaccharides (Glc-Gal-Rha-Man-Fuc) could be integrated into the engineered strains to promote the biosynthesis of targeted glycans. Subsequently, the engineered strains can be used as chassis cells whose synthesis capability of different serotypes could be further designed by specific exogenous GTs. We found a different situation when the same analysis was applied to the K antigen (Fig. 6B). With only 10 sugars involved in the K antigen, the combination of the four most common sugars (Glc-Gal-Rha-Man) covers 54% of the serotypes, and the addition of two sugars (Rib-Kdo) only increases the proportion to 80%. Therefore, such chassis cell design through serotype coverage may not be practical for K antigens.

### Sugar biosynthetic pathway

**Fig. 7:**
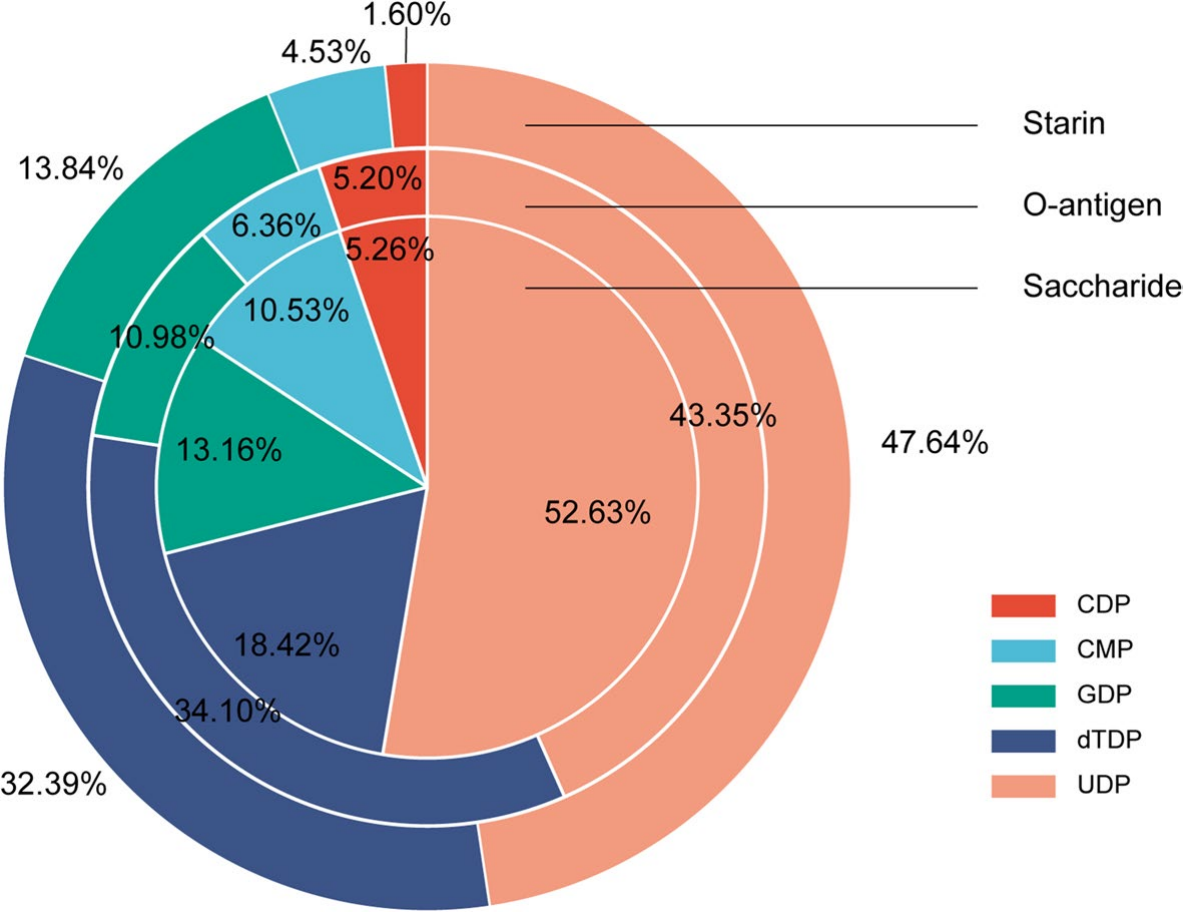
Nucleotide sugar forms in the sugar biosynthetic pathway. We screened for antigens and strains that have been found to have this pathway. The strain loop represents the frequency of this nucleotide sugar form at the strain differentiation level. O antigen loop represents the frequency of this nucleotide-sugar form at the O antigen differentiated level. Saccharide pie represents the frequency of this nucleotide-sugar form at the product differentiation level. Abbreviations: *CDP* Cytidine diphosphate, *CMP* Cytidine monophosphate, *GDP* Guanosine diphosphate, *dTDP* Deoxythymidine diphosphate, *UDP* Uridine diphosphate

Certain sugars may be difficult to assimilate from the environment. Therefore, sugar biosynthesis pathways that use common simple sugars to produce the required sugars for antigen synthesis are also important. A total of 38 pathways are included in this study, each with detailed step-by-step information, including substrates, products, enzymes, and involved serotypes and strains. Glucose plays an important role in the biosynthesis of various sugars because the initial precursors of 32 pathways are its derivatives (14 pathways for “glucose-1-phosphate”, 17 for “N-acetylglucosamine-1-phosphate”, and one for “UDP-GlcNAc”). As glucose is the most common monosaccharide in organisms with steady content, it is often used as the metabolic precursor of various sugars. Fructose-6-phosphate is an important intermediate in glycolysis and thus can effectively ensure the precursor supply involved in four pathways [32]. In addition, there are two initial precursors, ribulose-5-phosphate and sedoheptulose-7-phosphate, which are mainly involved in specific biosynthesis pathways. When viewed from the perspective of activated monosaccharides used for SP synthesis, the uridine diphosphate (UDP-) and deoxythymidine diphosphate (dTDP-) sugars were the most common forms, while cytidine monophosphate (CMP-), cytidine diphosphate (CDP-), and guanosine diphosphate (GDP-) sugars also accounted for a small proportion (Fig. 7). These synthetic pathways are the main sources of several sugars for bacteria and are, therefore, important targets for constructing chassis cells.

### Database structure and usage

A database was constructed based on the collected data to store and display the information centrally. The EcoSP database (http://ecosp.dmicrobe.cn/) is a manually curated resource that catalogs majority of the reported *E. coli* SPs. This information comprises three sections: GT, antigen (O antigen and K antigen), and pathway.

The “antigen” section includes two SP antigens of *E. coli*. The page of each O antigen serotype displays the information about glycosidic bonds, carbohydrate chemical structure, GT, antigen-synthesis gene cluster, and their corresponding strains (Figure S3). The K antigen page is similar, but no corresponding strain exists, this is due to the inconvenient identification of K antigen in general laboratories [33]. Therefore, we have added a separate search page to facilitate the exploration of the entries by the designated antigen type or strain (Figure S4). Searching for a serotype antigen will redirect the user to an antigen page, while searching for strains will display the strain-specific antigen information and GT information.

The “glycosyltransferase” section details the enzyme name, the relation to serotype, linkage, donor, acceptor, linkage type, and annotation for the enzyme (Figure S5). The user-provided keyword will be searched in every field, and a secondary search is also supported.

The “pathway” section contains information about the sugar biosynthesis pathway collected (Figure S6). To remove redundancy, we combined the same intermediate steps in different pathways into the same reaction label. The serotypes and strains involved were added to detect whether the target serotype or strain has the specific sugar biosynthesis capacity. In addition, this section integrates an interactive search tool to help users filter by keywords in real time.

### Performance of analysis tools

Based on the information collected in this study, we built the tools to perform serotype prediction and GT annotation analysis on the genome sequence of *E. coli* and closely related species. This section is also loaded into EcoSP as a web page. We tested the performance of these tools with several genome sequences.

**Fig. 8:**
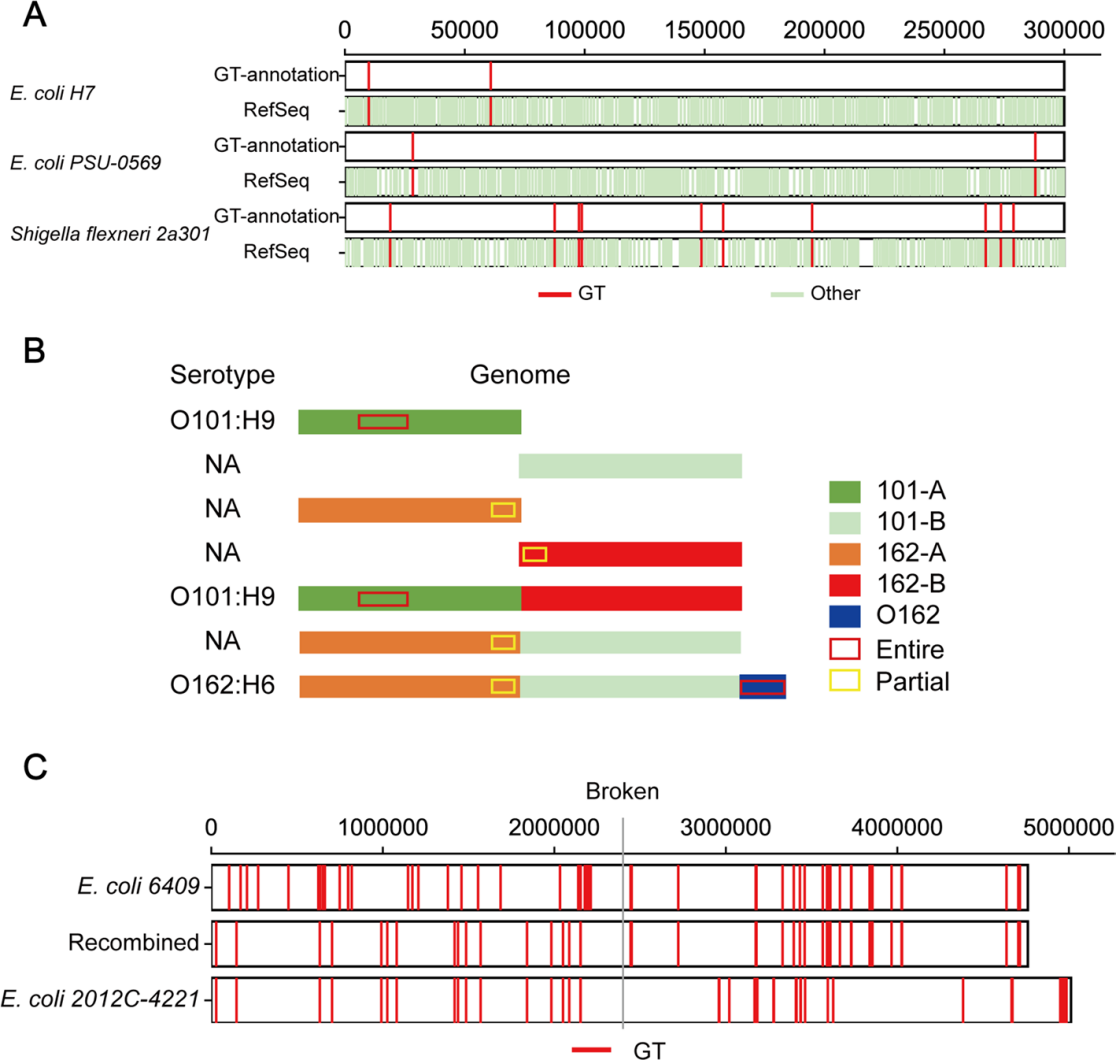
Performance of analysis tools. (**A**) Serotype prediction and GT annotation of various strains. For easy viewing, only the first 300 kb are shown. Two sequences are shown for each strain, including the analysis results and the information stored in the RefSeq database. (**B**) Serotype prediction in artificial genomes. The genome sequence of O101 strain was divided into fragments 101A (anterior) and 101B (posterior). The genome sequence of O162 strain was divided into fragment 162A (anterior) and 162B (posterior). The antigen synthesis gene cluster of O162 (denoted by O162) was also involved in the predictive analysis. Antigen synthesis gene clusters are marked in rectangular form, with red and yellow representing entire and partial gene clusters, respectively. (**C**) GT prediction effect of sequence with incomplete antigen. The recombined genome contains the anterior part of *E. coli 2012C-4221* and the posterior part of *E. coli 6409*

Since the sequences uploaded by users for GTs annotation analysis might be not deposited in the database, we simulated three different scenarios: a genome sequence of reported *E. coli* strain already indexed by our database (*E. coli H7* strain), a genome sequence of an unindexed *E. coli* strain (*E. coli PSU0569* strain), and an indistinguishable close-related non-*E. coli* strain (*Shigella flexenri 2a301* strain). The results (Fig. 8A) showed that GT annotation is correct as we expected and independent on genome sequences uploaded by different users. All of them could be annotated for GTs, and their serotypes were predicted. The results contain gene name, gene locus, GT name, glycosidic bond, donor, and acceptor saccharides. GT annotation in both included and unincluded strains was consistent with their information in the RefSeq database. Some GT prediction results from the proximal strain might slightly deviate at the start or end sites, but their locations roughly correspond. In addition, the analysis results are also accompanied by a serotype prediction in the form of O antigen serotypes, such as O107.

Considering that these genomes have been identified and included in NCBI, and new clinical isolates or artificial strains may have different mutations or recombination, we used several designed genome sequences to conduct a simulation test. The first analysis was performed with a disordered genome. The genome sequence of *E. coli 6409* (O101, GCA_000814145.2) was split into fragments every 200 kb (all GT genes were not interrupted), and then, fragments were randomly assembled into an artificial genome. GT annotation of this artificial genome sequence matched correspondingly after the recombination and could still accurately predict the previous serotype (Figure S7, Table S1-2). This suggests that translocations that do not affect relevant genes do not affect serotype prediction. In addition, partial genomes and heterologous recombinant genomes were also analyzed (Fig. 8B). Because of the similarity between O101 and O162, we performed a combined analysis with the genome sequence from serotype O162 (*E. coli 2012C-4221*, GCA_003018235.1) and *E. coli 6409* (O101). First, those two sequences were split at 2.4 Mb to create four partial genomes (101A, 101B, 162A, and 162B). Second, partial genomes were assembled to generate two heterologous recombinant genomes (101A-162B and 162A-101B). In these partial genomes, 101A could be predicted as the serotype (O101), while the other three partial genomes could not. For the heterologous recombinant genomes, the 101A-162B recombinant sequence could be predicted as serotype O101, while 162A-101B failed. To verify the sensitivity, we added a reference O162 antigen gene cluster to 162A-101B, and the sequence could be predicted as the serotype (O162). This suggests that the corresponding antigen-synthesis gene cluster is sufficient for serotype prediction and that recombination between different strains (or perhaps just DNA contamination) has no effect.

In addition, GT prediction can also be performed for genomes that fail to predict serotypes. For example, GT prediction was performed on sequence 162A-101B to obtain the GT loci, which were compared with the annotated information of the original sequence (Fig. 8C). Before and after the split site, recombinant sequence prediction results correspond to their original sequence's annotation. This indicates that the prediction of GT is completely independent, not affected by the whole genome composition, and the prediction results have high fidelity. Based on this, we were able to predict GTs in other bacteria. For example, we could predict the potential GTs in the genome sequence (GCA_008632635.1) of *Acinetobacter baumannii K09-14* (Table S3).

The results could be exported as tables, which can facilitate subsequent sorting. Given that the analysis of genome annotations is time-consuming, users can choose to leave an email address to receive a notification along with a result file when the analysis is complete. These two analyses can discover GTs in the target strain and thus have important implications for constructing our chassis cells and synthesizing the desired antigens using the chassis cells with several specific GTs. With these analysis tools, in-silico validation for whether the designed strain can produce the desired antigen also becomes possible.

## Conclusions and perspectives

We manually curated a relation database for *E. coli* SPs. Summarizing the data, we found similarities and differences between O and K antigens. Most of the sugars in the O antigen belong to hexose, and the main type is D- pyranose. For the K antigen, the situation is similar, except that there are significantly fewer types of sugars compared to the O antigen. This may be why the number of O antigens is larger than that of K antigens. Connections between Glc, Gal, Man, or Rha are the most common in both antigens, but the connection of Rib-Kdo is somewhat important in K antigens. In both O antigen and K antigen, the C1 carbon of the donor is the main link site, and it is usually connected to the receptor's C2, C3, or C4 carbon. Glc and its derivatives are mainly used as substrates for synthesizing the specific sugar, and UDP- or dTDP- activation is the major form in the synthesis process. This information led us to think about whether we could engineer desired strains. The genome of the engineered strain should contain at least the important monosaccharide synthesis pathways and some of the most common GTs, and thus have sufficient potential to synthesize target antigens with less and cheaper common sugars supplied during fermentation. Based on this consideration, we are also trying to construct the engineered strain and the corresponding GT gene library.

Several databases related to SPs have been built, but each has advantages and disadvantages. KEGG and MetaCyc are classical databases for biological research that have recorded many metabolic pathways in *E. coli *[27, 34]. Nevertheless, in-depth knowledge of a particular pathway is necessary to obtain the corresponding information accurately. Some databases, such as GTDB and CSDB, focus on glycosylation and contain multiple information on GT but have limited information about enzymes [28, 35]. These databases contain a variety of GTs from different species, not only GTs related to SP synthesis.

On the contrary, the ECODAB database collects only antigen-related information from the perspective of the O antigen. Its information on antigen structure is relatively complete but lacks information on GT or gene clusters [36]. Similarly, EK3D also mainly included structure information for a variety of K antigens, which are also classified according to their synthetic pathways [37]. However, these databases still have some drawbacks: i) lack of integration of O antigen and K antigen information, and the unavailable quick browsing to retrieve accurate synthetic gene cluster sequence information; ii) the antigen structure information is not organized with the monosaccharide or polysaccharide synthesis pathway information; iii) inability to quickly retrieve the serotypes of published *E. coli* genomes and make a prediction for unknown genomes. In general, they tend to be more of the chemical structure, and don't go far enough in the biological direction.

In contrast, EcoSP is a platform dedicated to *E. coli* SP research. In general, this database only focuses on the information related to SPs. It combines multiple relevant pieces of information so that the results are sufficient but not redundant. The advantage of this design is that researchers from different fields can easily use the database without expertise in SPs. We hope EcoSP will effectively facilitate gene mining and promote the construction of various polysaccharide synthesis engineered strains.

In the future, it is envisaged that the expanding data will provide more insights into synthetic pathways and antigen types. EcoSP is expected to regularly incorporate up-to-date information from the latest publications to ensure accuracy and coverage for representative antigens and GTs.

## Supplementary Information

**Additional file 1: Supplementary figures.**

**Additional file 2: Supplementary table 1.**

**Additional file 3: Supplementary table 2.**

**Additional file 4: Supplementary table 3.**

## Data Availability

We obtained the genome sequences of all strains (more than 12,000 strains) from the *Escherichia coli* genome page (https://www.ncbi.nlm.nih.gov/genome/browse/#!/prokaryotes/167/) in NCBI database, and screened 7741 strains for the construction of the information network. The original genome sequences used for test were also obtained from the NCBI database (*E. coli H7*, GCA_001900455.1; *E. coli PSU0569*, GCA_012214065.1; *Shigella flexneri 2a str. 301*, GCA_000006925.2; *E. coli 6409*, GCA_000814145.2; *E. coli 2012C-4221*, GCA_003018235.1; *Acinetobacter baumannii K09-14*, GCA_008632635.1). The relevant information involved in this study has been integrated into the EcoSP database (http://ecosp.dmicrobe.cn/).

## Abbreviations

SP: Surface polysaccharides
*E. coli*: *Escherichia coli*
GT: Glycosyltransferase
LPS: Lipopolysaccharide
CPS: Capsular polysaccharide
BLAST: Basic Local Alignment Search Tool
Glc: Glucose
Fuc: Fucose
Rha: Rhamnose
Gal: Galactose
Man: Mannose
Rib: Ribitol
Kdo: 3-Deoxy-d-manno-oct-2-ulosonic acid
Neu: N acetylneuraminic acid
Tal: Talose
UDP: Uridine diphosphate
dTDP: Deoxythymidine diphosphate
CMP: Cytidine monophosphate
CDP: Cytidine diphosphate
GDP: Guanosine diphosphate

## References

[1] Jann K, Jann B (1987). Polysaccharide antigens of Escherichia coli. Rev Infect Dis.

[2] Luthje P, Brauner A (2014). Virulence factors of uropathogenic E coli and their interaction with the host. Adv Microb Physiol..

[3] Sarkar S, Ulett GC, Totsika M, Phan MD, Schembri MA (2014). Role of capsule and O antigen in the virulence of uropathogenic Escherichia coli. PloS one..

[4] Bos MP, Robert V, Tommassen J (2007). Biogenesis of the gram-negative bacterial outer membrane. Annu Rev Microbiol.

[5] Silhavy TJ, Kahne D, Walker S (2010). The bacterial cell envelope. Cold Spring Harb Perspect Biol..

[6] Raetz CR, Whitfield C (2002). Lipopolysaccharide endotoxins. Annu Rev Biochem.

[7] Liu B (2020). Structure and genetics of Escherichia coli O antigens. FEMS Microbiol Rev.

[8] Pawlak, A. *et al.* Salmonella O48 Serum Resistance is Connected with the Elongation of the Lipopolysaccharide O-Antigen Containing Sialic Acid. Int J Mol Sci 2017; 18. 10.3390/ijms18102022. 10.3390/ijms18102022 PMC5666704 28934165

[9] Torraca V (2019). Shigella sonnei infection of zebrafish reveals that O-antigen mediates neutrophil tolerance and dysentery incidence. PLoS Pathog.

[10] Whitfield C (2006). Biosynthesis and assembly of capsular polysaccharides in Escherichia coli. Annu Rev Biochem.

[11] Corbett D, Roberts IS (2008). Capsular polysaccharides in Escherichia coli. Adv Appl Microbiol.

[12] Abreu AG, Barbosa AS (2017). How Escherichia coli Circumvent Complement-Mediated Killing. Front Immunol.

[13] Mobley, H. L. & Alteri, C. J. Development of a Vaccine against Escherichia coli Urinary Tract Infections. Pathogens 2015; 5. 10.3390/pathogens5010001. 10.3390/pathogens5010001 PMC4810122 26729174

[14] Huttner A (2017). Safety, immunogenicity, and preliminary clinical efficacy of a vaccine against extraintestinal pathogenic Escherichia coli in women with a history of recurrent urinary tract infection: a randomised, single-blind, placebo-controlled phase 1b trial. Lancet Infect Dis.

[15] Frenck RW (2019). Safety and immunogenicity of a vaccine for extra-intestinal pathogenic Escherichia coli (ESTELLA): a phase 2 randomised controlled trial. Lancet Infect Dis.

[16] Bundle D (2016). Antibacterials: A sweet vaccine. Nat Chem.

[17] Kong L (2016). An antibacterial vaccination strategy based on a glycoconjugate containing the core lipopolysaccharide tetrasaccharide Hep2Kdo2. Nat Chem.

[18] Wang L, Wang Q, Reeves PR (2010). The variation of O antigens in gram-negative bacteria. Subcell Biochem.

[19] Kalynych S, Morona R, Cygler M (2014). Progress in understanding the assembly process of bacterial O-antigen. FEMS Microbiol Rev.

[20] Han W (2012). Defining function of lipopolysaccharide O-antigen ligase WaaL using chemoenzymatically synthesized substrates. J Biol Chem.

[21] Ruan X, Loyola DE, Marolda CL, Perez-Donoso JM, Valvano MA (2012). The WaaL O-antigen lipopolysaccharide ligase has features in common with metal ion-independent inverting glycosyltransferases. Glycobiology.

[22] Willis LM, Whitfield C (2013). Structure, biosynthesis, and function of bacterial capsular polysaccharides synthesized by ABC transporter-dependent pathways. Carbohydr Res.

[23] Bertani, B. & Ruiz, N. Function and Biogenesis of Lipopolysaccharides. EcoSal Plus 2018; 8. 10.1128/ecosalplus.ESP-0001-2018. 10.1128/ecosalplus.esp-0001-2018 PMC6091223 30066669

[24] Parks DH, Imelfort M, Skennerton CT, Hugenholtz P, Tyson GW (2015). CheckM: assessing the quality of microbial genomes recovered from isolates, single cells, and metagenomes. Genome Res.

[25] Jain C, Rodriguez RL, Phillippy AM, Konstantinidis KT, Aluru S (2018). High throughput ANI analysis of 90K prokaryotic genomes reveals clear species boundaries. Nat Commun.

[26] Joensen KG, Tetzschner AM, Iguchi A, Aarestrup FM, Scheutz F (2015). Rapid and Easy In Silico Serotyping of Escherichia coli Isolates by Use of Whole-Genome Sequencing Data. J Clin Microbiol.

[27] Caspi R (2020). The MetaCyc database of metabolic pathways and enzymes - a 2019 update. Nucleic Acids Res.

[28] Parks DH (2022). GTDB: an ongoing census of bacterial and archaeal diversity through a phylogenetically consistent, rank normalized and complete genome-based taxonomy. Nucleic Acids Res.

[29] Bochkov AY, Toukach PV (2021). CSDB/SNFG Structure Editor: An Online Glycan Builder with 2D and 3D Structure Visualization. J Chem Inf Model.

[30] Hyatt D (2010). Prodigal: prokaryotic gene recognition and translation initiation site identification. BMC Bioinformatics.

[31] Kanayama A (2015). Enterohemorrhagic Escherichia coli outbreaks related to childcare facilities in Japan, 2010–2013. BMC Infect Dis.

[32] Cao Y, Li M, Xia Y (2011). Mapmi gene contributes to stress tolerance and virulence of the entomopathogenic fungus. Metarhizium acridum J Invertebr Pathol.

[33] Fratamico PM (2016). Advances in Molecular Serotyping and Subtyping of Escherichia coli. Front Microbiol.

[34] Kanehisa M, Furumichi M, Tanabe M, Sato Y, Morishima K (2017). KEGG: new perspectives on genomes, pathways, diseases and drugs. Nucleic Acids Res.

[35] Egorova KS, Toukach PV (2017). CSDB_GT: a new curated database on glycosyltransferases. Glycobiology.

[36] Lundborg M, Modhukur V, Widmalm G (2010). Glycosyltransferase functions of E. coli O-antigens. Glycobiology..

[37] Kunduru BR, Nair SA, Rathinavelan T (2016). EK3D: an E. coli K antigen 3-dimensional structure database. Nucleic Acids Res..

