# Supplementary material for: Bioinformatic analysis of structures and encoding genes of Escherichia coli surface polysaccharides sheds light on the heterologous biosynthesis of glycans
Source: BMC Genomics. 2023 Apr 4;24:168. doi: 10.1186/s12864-023-09269-6 (PMC10072801; doi:10.1186/s12864-023-09269-6)
Supplement: Supplementary file 1 — Additional file 1: Supplementary figures. [file 12864_2023_9269_MOESM1_ESM.docx]

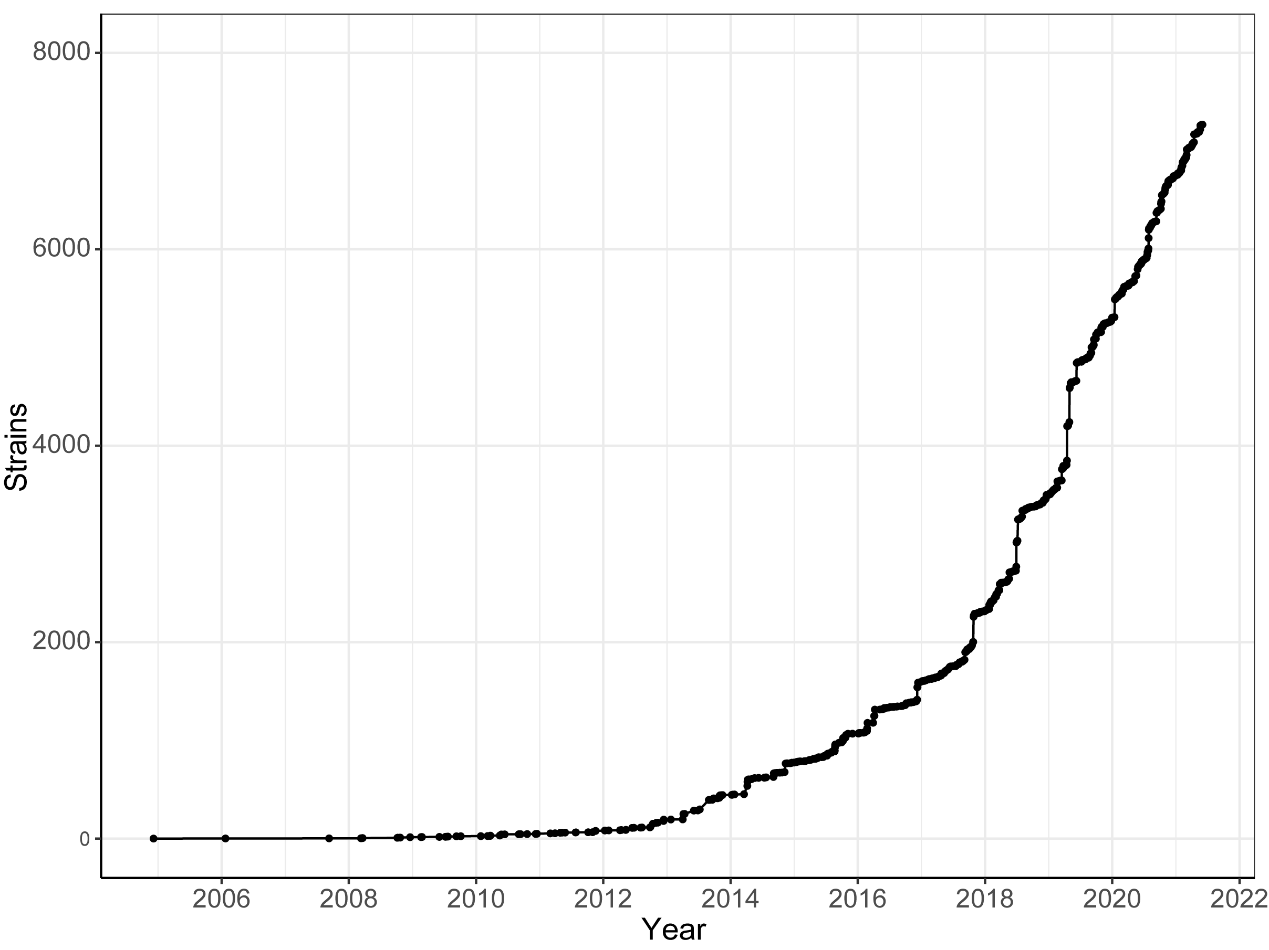


**Figure S1. Submission date of collected genomes.** The date that genome is submitted to NCBI, or updated, which is taken from its descriptive information.


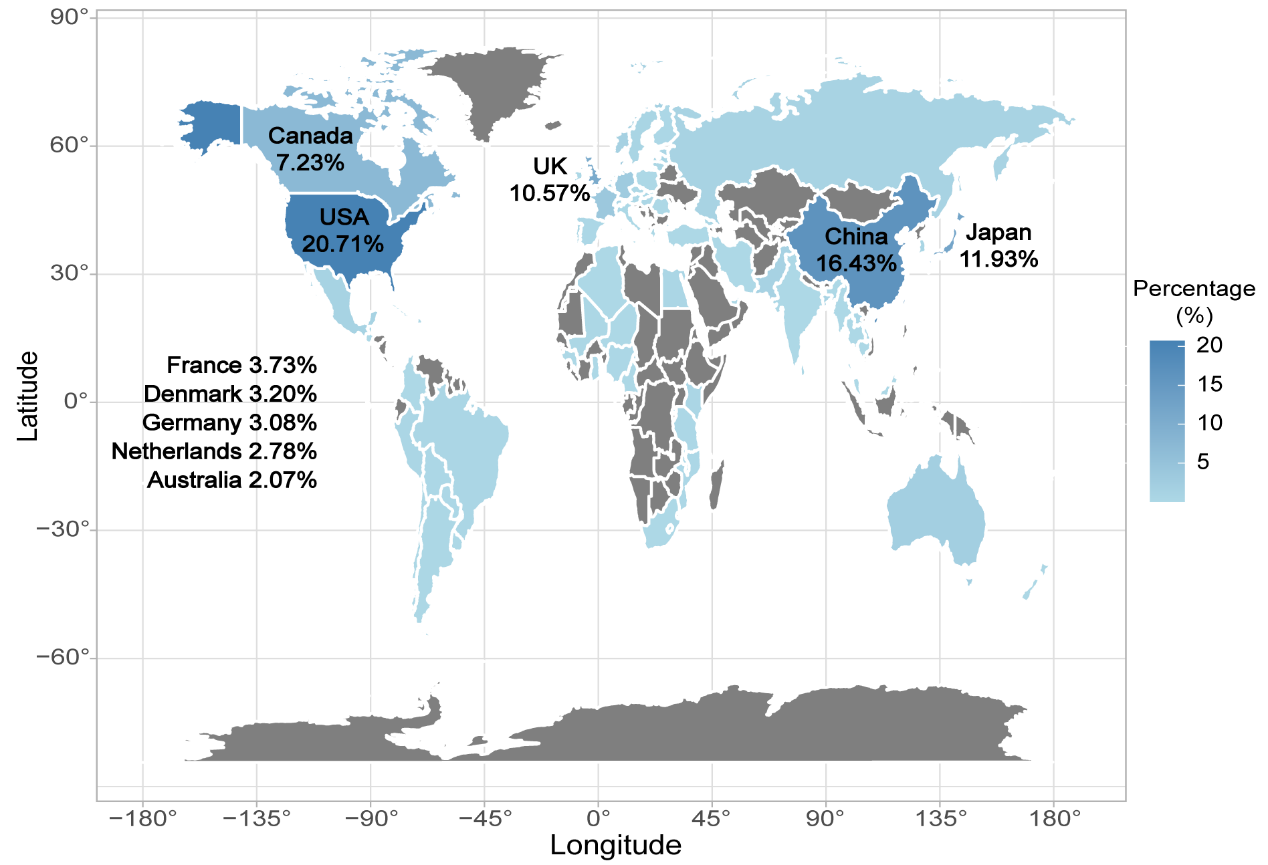


**Figure S2. Geographical origin of collected strains.** Geographic area where each strain was isolated, which is taken from its descriptive information.


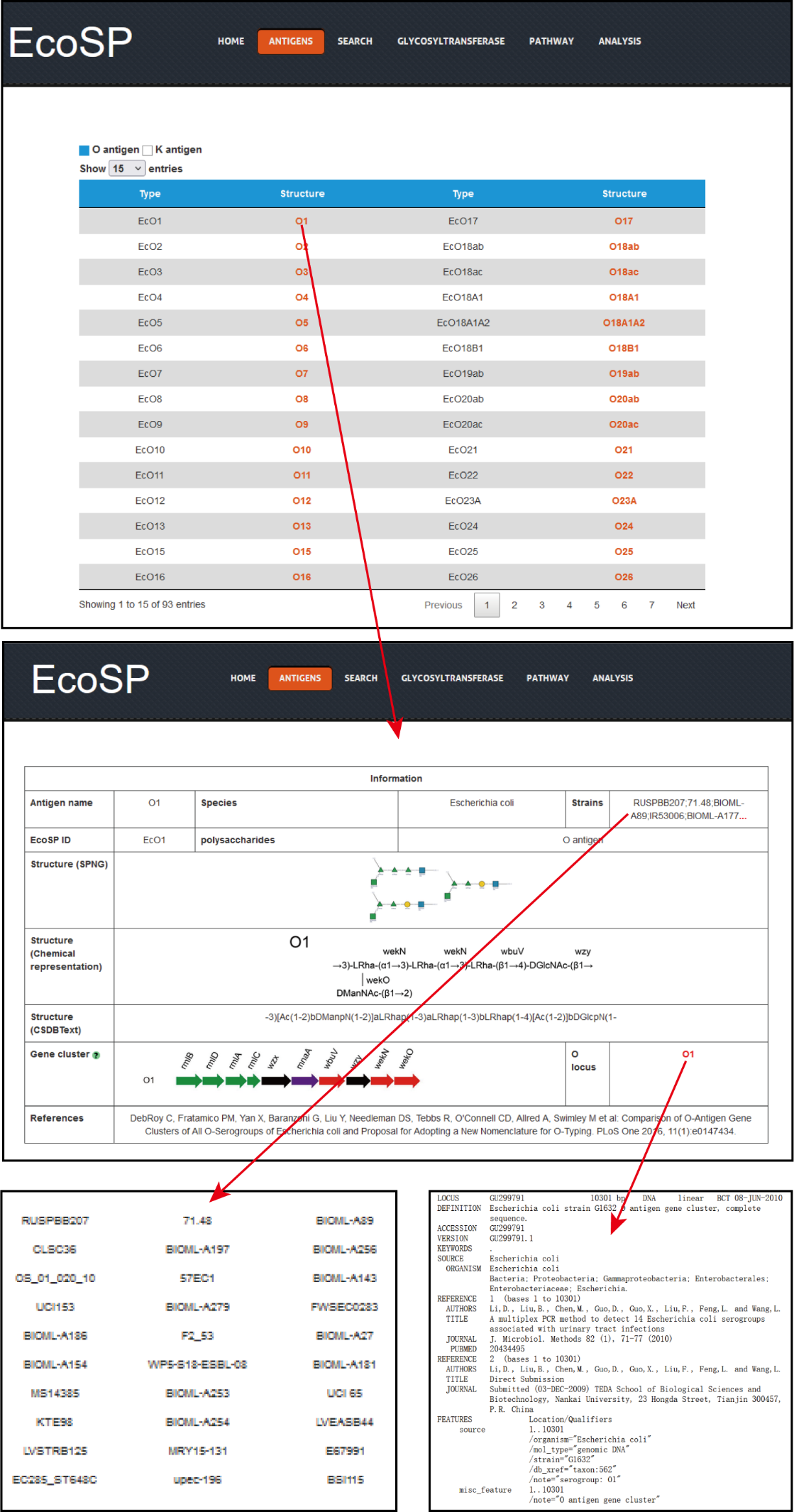


**Figure S3. Screenshot of relevant pages in antigen section.** This section is based on an antigen overview page, which assembles the hyperlink for each specific antigen page, and further links to the gene cluster information page and a strain information popup.


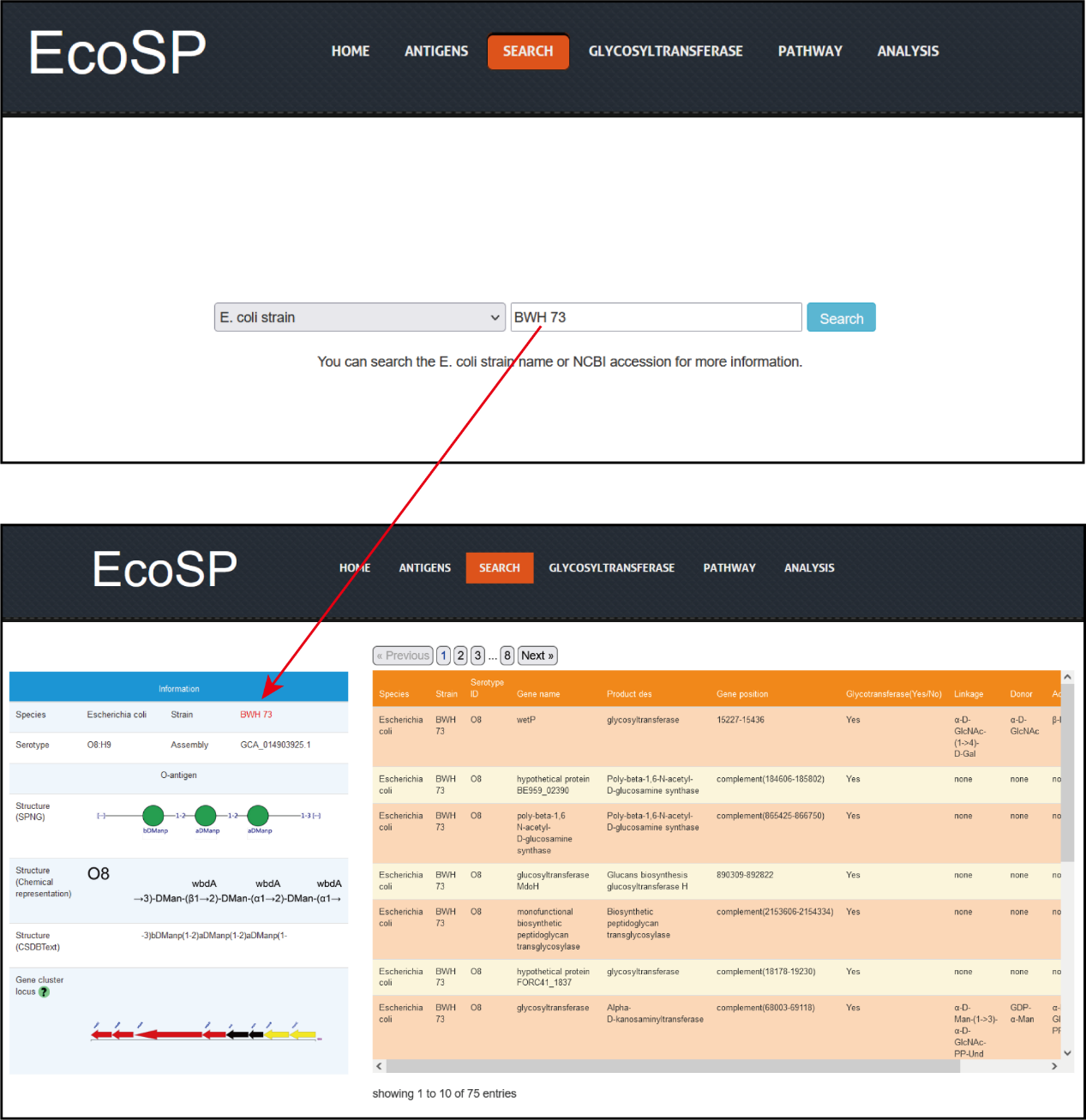


**Figure S4. Screenshot of relevant pages in search section.** This section is based on a search engine, which can jump to the corresponding antigen page by searching the antigen, or search the strain name to display their information included in this database.

**
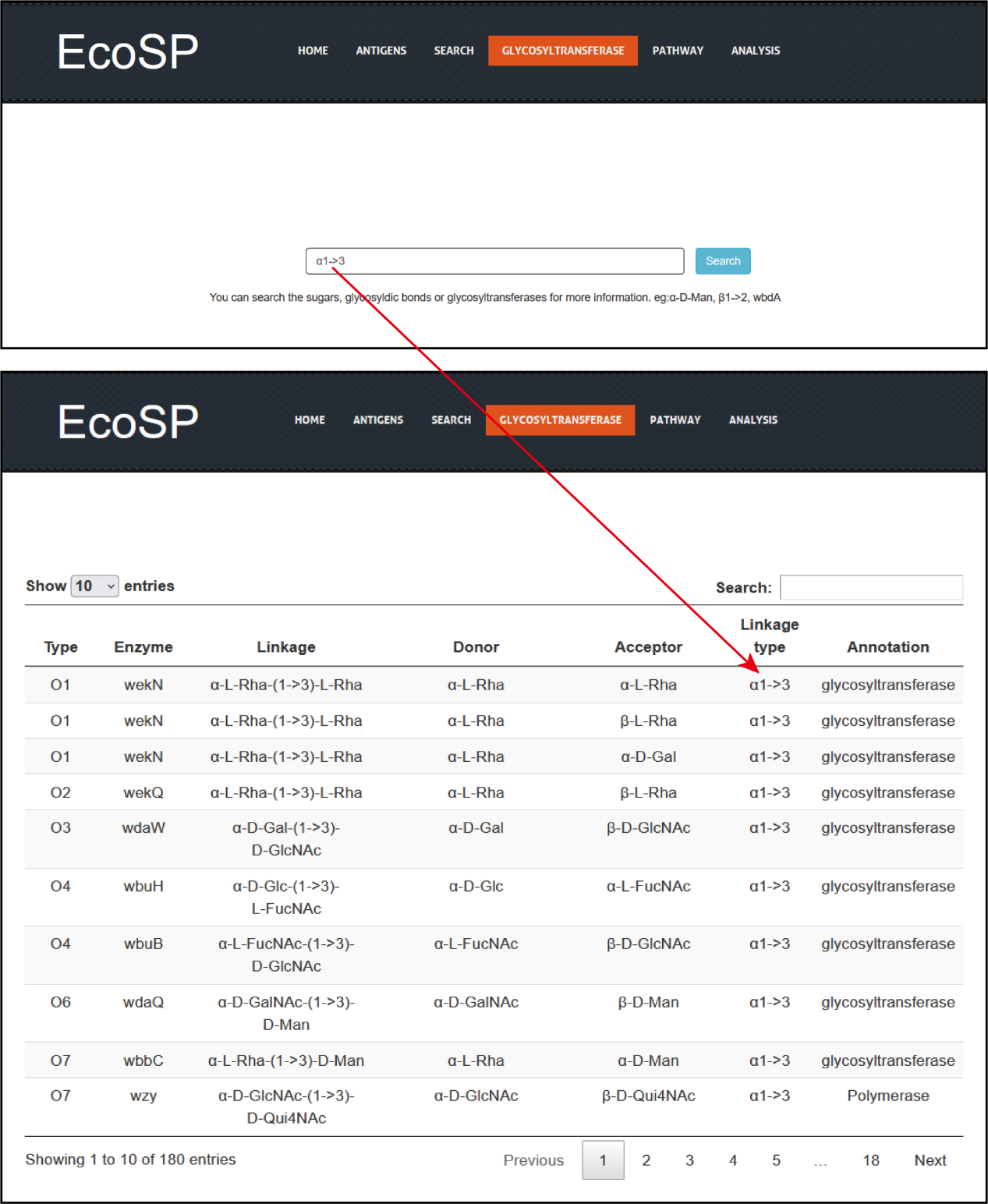
**

**Figure S5. Screenshot of relevant pages in glycosyltransferase section.** This section is based on a search engine, which can search for GT related information, such as GT name, sugar, glycoside bond, to obtain the corresponding information.

**
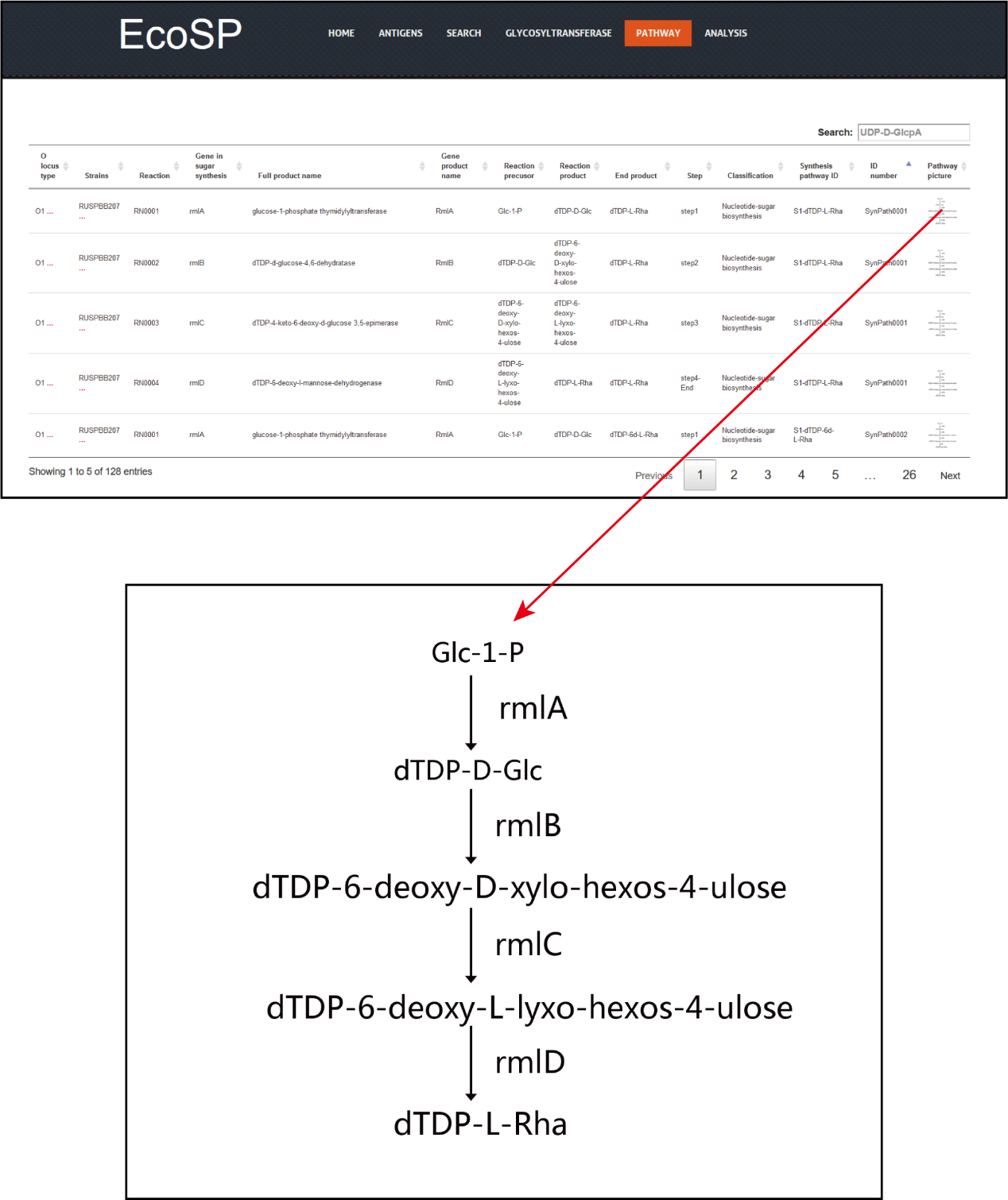
**

**Figure S6. Screenshot of relevant pages in pathway section.** This section is displayed on a page with pop-ups that show which antigens or strains are involved in the reaction. Each reaction has a metabolic diagram of the whole pathway.

**
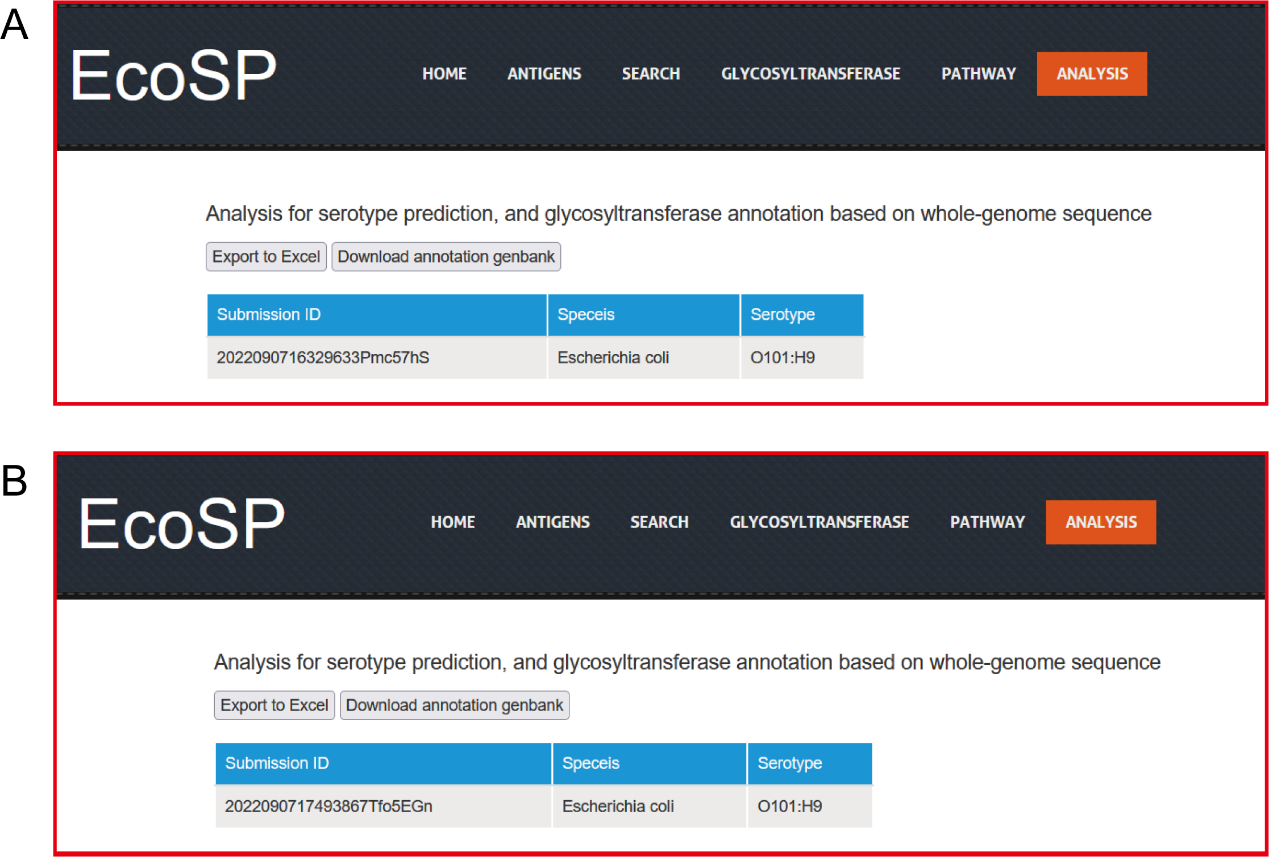
**

**Figure S7. Serotype prediction before and after genome sequence recombination.** (A) Serotype prediction of original genome sequence. (B) Serotype prediction of recombinational genome sequence.
